# How Emotional Contagion among Teachers Affects the Relationship between Transformational Leadership and Team Cohesion

**DOI:** 10.3390/bs13080685

**Published:** 2023-08-16

**Authors:** Giulia Paganin, Lorenzo Avanzi, Dina Guglielmi, Carlos-María Alcover, Greta Mazzetti

**Affiliations:** 1 Department of Medical and Surgical Science, University of Bologna, 40126 Bologna, Italy; 2 Department of Psychology and Cognitive Sciences, University of Trento, 38068 Rovereto, Italy; 3 Department of Educational Science, University of Bologna, 40126 Bologna, Italy; 4 Department of Psychology, Rey Juan Carlos University, 28922 Madrid, Spain

**Keywords:** transformational leadership, teachers, emotional contagion, organizational identification, team cohesion, school

## Abstract

Teachers and educators are experiencing turmoil under the drastic changes in educational practices caused by the COVID-19 pandemic. According to research, transformational leaders effectively facilitate organizational change by fostering teachers’ sense of belonging and boosting social identity in their team members, which can result in better team well-being via higher team cohesion. Recently, research has increasingly explored the role of emotional contagion and its relationship with leadership. Accordingly, the current study aims to delve deeper into the role of emotional contagion in linking transformational leadership to cohesion among teachers in the school setting. To this purpose, 581 teachers from northern Italy filled out a self-report questionnaire (72.1% female, M_age_ = 47.06, and SD_age_ = 11.42). A moderated mediation model was tested to assess the mediating role of organizational identification in the relationship between transformational leadership and team cohesion and how emotional contagion may moderate this association. The obtained results provided support to the hypothesized model. Overall, the present study corroborates the critical role of school principals’ behavior in fostering greater organizational identification among teachers, which is associated with better team cohesion. This study constitutes an early attempt to gain more insight into the role of emotional variables in explaining the influence of leadership behavior.

## 1. Introduction

The teaching profession has traditionally been considered particularly demanding because of the need to operate in an ever-changing work environment, to deal with far-reaching political and social changes, and to perform cognitive and emotional release tasks [1]. In addition, the COVID-19 pandemic has led to numerous reforms in teachers’ pedagogical practices worldwide in recent years [2]. Several studies in the educational context have examined the effects of different leadership styles on organizational outcomes. 

These studies support the assumption that transformational leaders can act as role models (e.g., [3,4]). By inspiring, encouraging, and supporting their followers, transformational leaders are able to increase their followers’ commitment to the organization, thus creating a high sense of belonging. 

Consequently, those who perceive a higher level of social identification may perceive a higher association with group-level outcomes, including cooperation and team cohesion [5,6]. Team cohesion is an important outcome, as it could be considered as a proxy for well-being, since it is postulated that belonging to a highly cohesive group can contribute significantly to the well-being of its members [7]. Moreover, it could be associated with a higher work performance [8,9].

The latest research on team cohesion underlines the need to broaden the list of variables studied as antecedents [10]. At the same time, researchers’ interest in the phenomenon of emotional contagion and its link with leadership has increased in recent years [11]. Thus, it may be interpreted as a key condition through which organizational behavior acts.

Despite extensive research on transformational leadership, more investigation is needed to fully comprehend its mediation and moderation mechanisms [12,13,14]. In addition, leadership has been typically studied as behavior directed toward individuals. From this perspective, new research is called for that also investigates the relationship with team outcomes (e.g., [15]).

**Figure 1:**
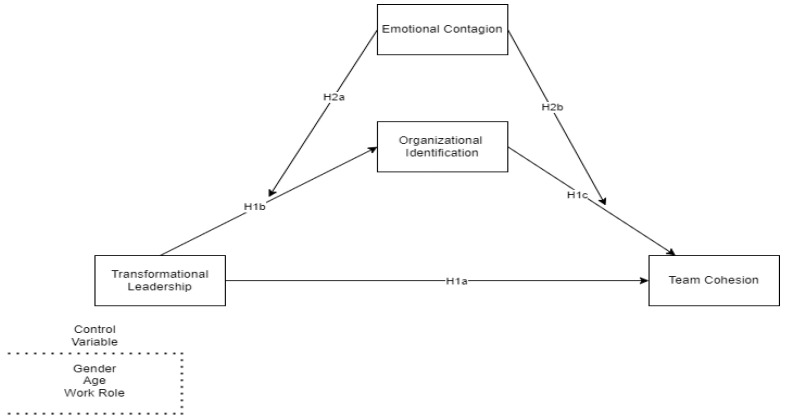
Hypothesized model.

Relying on this background and given the need to investigate boundary conditions that influence the impact of transformational leadership, this study aims to deepen the association between transformational leadership and team cohesion by analyzing the mediating role of organizational identification. Furthermore, this study seeks to clarify to what extent followers’ susceptibility to emotional contagion could affect the strength of the previous relationship, by interacting with transformational leadership and organizational identification (see Figure 1). 

### 1.1. Theoretical Background

According to Bass [16], transformational leadership combines four primary dimensions: idealized influence, inspirational motivation, individualized consideration, and intellectual stimulation. These behaviors that characterize transformational leadership have been proven to have a significant and progressive influence over subordinates in educational contexts [17], for example, in terms of positive relationships with school performance [18].

A noteworthy meta-analysis proposed by Burke [19] pointed out that the development of team cohesion resulted from the transformational emphasis on shared higher-order needs and shared ideals or visions. Indeed, researchers have proposed that an environment that encourages increased contact among team members will promote team cohesiveness [20].

Team cohesion was defined as “a dynamic process that is reflected in the tendency for a group to stick together and remain united in the pursuit of its instrumental objectives and/or for the satisfaction of member affective needs” [21]. It has consistently been linked to team effectiveness and performance [8,9], as well as with the well-being of individual team members [7,22]. Many other variables can influence the perception of group cohesion. In fact, multi-level studies have shown that individual-, team-, and organization-level factors can affect team-level outcomes [10,23]

Even though most of the studies conducted to date gave relevance to individual-level outcomes of transformational leadership, this tendency does not imply a limited relevance that could be attributed to group-level research [24]. 

Jung and Sosik [25] suggested that the underpinning mechanism behind the interplay between transformational leadership and cohesion lies in the leaders’ function of pointing out the relevance of cooperation and realigning followers’ values. Furthermore, Muppidathi and Krishnan [26] adopted a different approach by connecting each transformational leadership component with group cohesiveness. 

Thus, the following hypothesis was developed:

**Hypothesis** **1a (H1a)**. *Transformational leadership is positively related to team cohesion*.

Organizational identification is described as the “feeling of uniqueness or belonging to the organization” [5]. Using the Social Identity Theory as a framework [27,28], organizational identity can be conceived as a particular instance of social identity, with organizational identification as the driving force behind this identity. This theory contends that humans develop their sense of self through joining social groups and appropriating the traits of such groups as their own [29]. People identify with a group primarily in order to boost their perception of their collective self-worth [30]. 

Organizational identification occurs when employees see themselves as part of their organization, leading them to align their interests and objectives with those of the organization. It predicts group-based behaviors and crucial attitudes and workplace behaviors [31,32]. 

Several previous studies have proved a positive relationship between leadership styles and organizational identification, especially concerning transformational leadership [33,34,35]. Inspiring, encouraging, and assisting followers, transformational leaders enhance employees’ dedication toward the organization [34], thus forging a greater sense of belonging and boosting the feeling of being part of something bigger [36]. Accordingly, transformational leaders succeed in connecting followers’ self-concept with the mission and the scope of the organization [37]. In turn, this higher connectedness may have positive outcomes at all levels of the organization in terms of higher individual working satisfaction and performance at an individual level [38], and team cohesion at a group level [39]. Transformational leaders build a supportive work environment that results in a setting that maximizes employees’ potential and helps them solve work-related challenges. Consequently, employees feel proud of the company and perceive a sense of belonging, strengthening their identification with the institution [34]. 

Consistent with previous studies, we hypothesize that:

**Hypothesis** **1b (H1b)**. *Transformational leadership will positively affect followers’ organizational identification*.

The Social Identity perspective supports the significant relationship between identification with proximal peer groups and well-being [40]. For example, Turner [6] suggests that as more individuals experience social identification, stronger will be the associations with group-level outcomes, including intrateam cohesion, cooperation, and altruism. In a work context, organizational identification has to do with a person’s sense of unity or belonging to the organization. Individuals are prompted to be engaged in activities congruent with their identity, to view themselves as part of a group, and thus to strengthen team cohesion and interactions [5]. Employees will therefore internalize organizational rules and goals more readily and perceive themselves as interchangeable with other members as they become more identified with the organization. Consequently, greater collaboration among co-workers ought to follow from this increased identification [41,42,43].

A strong organizational identification should lead employees to see themselves as more similar to other colleagues, and more related to each another, fostering shared values and norms into the groups. Decoster et al. [39] found a positive correlation between organizational identification and group cohesion in a small sample of employees. 

Moreover, to increase followers’ intrinsic desire to execute their jobs, transformational leaders offer ideological justifications that connect followers’ identities to the collective identity of their work group or organization [44]. Consequently, priming followers’ collective levels of self-identity would raise the likelihood that they would act cooperatively to support the organizational mission and goals rather than their objectives [45]. Garcia-Guiu Lopez et al. [46] tested a similar mediational model in security teams, in which authentic leadership indirectly and positively correlated with group cohesion, through its relationship with group identification. Thus, we developed the following hypothesis:

**Hypothesis** **1c (H1c)**. *Organizational identification will be positively related to teachers’ team cohesion*.

**Hypothesis** **1d (H1d)**. *Organizational identification mediates the relationship between transformational leadership and team cohesion. Specifically, transformational leadership is positively related to organizational identification, which, in turn, reports a positive relationship with team cohesion*.

### 1.2. Moderating Effect of Emotional Contagion 

Emotional contagion is crucial to our knowledge of organizational behavior because it may be a key moderator mechanism for collective emotion arising through conscious and unconscious emotional, social influence [11,47]. Emotional contagion is defined as the automatic imitation and synchronization of facial expressions, voices, postures, and movements with others. This phenomenon leads to behavioral alignment through emotional blending with those around us [48] and includes both positive and negative emotions. People with high emotional contagion are naturally better at expressing their emotions, which can significantly impact others [49], even if some people are more prone to it than others [50]. Although interest in emotional contagion is growing, a few studies have considered its role concerning the variables studied in our research. We may infer a contagious process also to a leadership setting, suggesting that followers can relate to and notice their leaders’ emotional displays and that leaders might use these to influence their workers [51]. 

According to Mindeguia and colleagues [52], transformational leaders can influence their followers’ emotions and behaviors by emotional contagion. A recent study considered the moderating effects of emotional contagion, including leaders’ emotional contagion and subordinates’ emotional susceptibility, in the relationship between transformational leadership and subordinates’ job involvement [51]. A further study investigating the positive association between emotional contagion and organizational identification empirically corroborated this relationship [53]. Concerning the relationship between emotional contagion and team cohesion, the few studies available are primarily addressed to sports contexts (e.g., [54]). However, a study performed in a workplace setting on a similar dimension (group positive affect), showed that a positive emotional climate within teams was associated with better team cohesion, as members felt a stronger commitment to the group goals [55]. In addition, previous research indicates that positive emotions are a strong predictor of workers’ well-being (e.g., beneficial emotional states), in addition to their intrinsic worth, and contribute to the promotion of beneficial organizational outcomes such as, but not limited to, teamwork, innovation, and job performance [56]. 

Still, the moderating effect of emotional contagion is left unclear and largely unexplored. 

A leader’s display of confidence and optimism, emphasizing followers’ accomplishments (transformational leadership), can foster a stronger bond between employees and their organization, leading to increased organizational identification. Emotional contagion plays a vital role in this process. For instance, positive attitudes and behaviors from a leader can elicit positive emotions in employees, triggering a favorable response from supervisors, creating a positive circle. Likewise, strong emotional contagion reinforces the relationship between identification and cohesion. A sense of belonging among employees enhances team cohesion as it promotes cooperative and collaborative behaviors. Emotional contagion facilitates emotional convergence among group members, enabling quick responses to coworkers’ signals and alignment with their emotions, further reinforcing the impact of organizational identification on subsequent attitudes and behaviors, such as employees’ cohesion. Thus, in line with the few results reported, we developed the following exploratory hypothesis:

**Hypothesis** **2a (H2a)**. *Emotional contagion will moderate the relationship between transformational leadership and organizational identification. We hypothesize that the positive association between transformational leadership and organizational identification will be more significant for employees with higher emotional contagion*.

**Hypothesis** **2b (H2b)**. *Emotional contagion will moderate the relationship between organizational identification and team cohesion. Specifically, we expect the positive association between organizational identification and team cohesion to be more significant for employees characterized by higher emotional contagion*.

**Hypothesis** **2c (H2c)**. *Emotional contagion will moderate the indirect path between transformational leadership and team cohesion via organizational identification*.

## 2. Method

### 2.1. Participants and Procedure

This survey is part of an assessment of well-being and stress risk as required by the Italian Law for the Prevention of Work-Related Stress (Legislative Decree 81/2008, “Testo Unico sulla Sicurezza”) and occurred between December 2020 and May. The online survey was delivered to 800 teachers and completed by 581 teachers at all grade levels in northern Italy (return rate 72.63%). Participants received an email asking them to access a specific website where they could answer the questionnaire by clicking on an anonymous link. In accordance with the standards for handling personal data set forth in the Italian Data Protection Act (Legislative Decree DL-196/2003), a cover letter was attached to the first page of the questionnaire explaining the scope and objectives of the study and emphasizing the privacy and anonymity of the participants. Consequently, it was assumed that participants had given their consent by completing the survey. According to relevant institutional and national criteria, the study did not require ethical approval because it complied with the most recent Declaration of Helsinki (World Medical Association, 2013), which sets forth ethical requirements for research. Because there was no treatment—including medical invasive diagnostics or procedures that caused psychological or social discomfort to participants—no additional ethical approval was required. The gender of participants was 72.5% female. A total of 31.2% of participants were between 40 and 49 years old. With respect to job role, 82.4% of the teachers indicated that they were secondary school teachers; 9.4% indicated that they were secondary school teachers; 5.7% indicated that they were support teachers; 1.7% indicated that they were elementary school teachers; and only 0.7% indicated that they were school leaders. On average, teachers reported working 25.78 (SD = 11.8) hours per week and working in the institution where they are serving on average for 7.9 (SD = 8.56) years. Finally, 59.9% of respondents reported having a full-time permanent contract, 20% having a full-time fixed-term contract, 10.3% having a part-time permanent contract, and 8.7% having a part-time fixed-term contract. The remaining 0.9% reported having another type of contract.

### 2.2. Measures

For all measures, respondents rated the item on a 5-point Likert scale, ranging from 1 = strongly disagree to 5 = strongly agree.

Transformational leadership was measured by a 7-item questionnaire developed by Carless and colleagues [57], adapted for the school context. Items included “The school principal communicates a clear and positive vision of the future”. Cronbach α coefficient for the scale was 0.94. 

Organizational identification was measured by a 6-item questionnaire validated in Italian by Manuti and Bosco [58], adapted for the school context. Items included “The achievements of the school I work for are my achievements”. Cronbach α coefficient for the scale was 0.85. 

Emotional contagion was measured using the 15-item questionnaire developed by Doherty [59], comprising 5 subscales covering different emotions: sadness (e.g., “If someone I’m talking with begins to cry, I get teary-eyed”), happiness (e.g., “Being with a happy person picks me up when I’m feeling down”), anger (e.g., “I clench my jaws and my shoulders get tight when I see the angry faces on the news”), love (e.g., “When I look into the eyes of the one I love, my mind is filled with thoughts of romance”), and fear (e.g., “Watching the fearful faces of victims on the news makes me try to imagine how they might be feeling”). Cronbach α coefficient for the overall scale was 0.86. 

Team cohesion was measured by a 4-item questionnaire [60]. Items included “When there is conflict on this team, the people involved usually talk it out and resolve the problem successfully”. Cronbach α coefficient for the scale was 0.83.

### 2.3. Strategy of Analysis

Data were analyzed using SPSS software (version 28, SPSS Inc., Chicago, IL, USA). First, we examined the normality, kurtosis, and skewness indices of the studied variables. To further evaluate the relationship between all study variables, we calculated mean values, standard deviations (SDs), Cronbach’s alpha, and bivariate correlation coefficients. We were able to determine magnitudes for “small” (0.10), “medium” (0.30), and “large” (0.50) correlation effects using Cohen’s criteria [61]. The hypothesized models, the simple mediation model and the further moderated mediation model are tested with the PROCESS macro (Model 4 and Model 58). The advantage of PROCESS is that it allows us to analyze the moderated mediation model by evaluating all path coefficients at once and examining the direct and indirect effects of the hypothesized model. We first ran a mediation model to examine the mediating role of organizational identification between transformational leadership and team cohesion.

The subsequent moderated mediation model also examines whether the mediated relationship between transformational leadership and team cohesion is moderated by emotional contagion. Two linear regression analyses serve as the basis for the moderated mediation model [62]. The independent variable, the moderator, and the interaction between the independent and moderating variables predict the mediator in the first regression analysis. In the second regression analysis, the independent variable, the moderator, their interaction, the first mediator, and the second mediator predict the dependent variable.

Prior to the analyses, the variables included in the hypothesized moderation effects (transformational leadership, organizational identification, and emotional contagion) were centered. Simple slope analyses were used to examine how the independent and moderating variables interacted [62,63]. Specifically, conditional effects were examined at low (1 SD below the mean), medium (mean), and high (1 SD above the mean) levels of emotional contagion. Based on 5000 bootstrap samples, we estimated indirect and moderating effects, including 95% bias-corrected confidence intervals (CIs). Finally, the single factor Harman test was used to examine common-method bias. According to the results, it does not seem to have a significant impact on the current study (only 24.48% of the covariance is explained by a single factor).

## 3. Results

### 3.1. Descriptive Statistics

Means, standard deviations, and correlations were computed for all study variables (Table 1). All significant relationships between the variables were in the expected direction. Furthermore, all scales reported an internal consistency (Cronbach’s alpha) above the recommended threshold of 0.70 [64].

### 3.2. Mediation and Moderation Effects

Regarding the mediated relationship assumed here, the hypothesized model was examined using model 4 from the PROCESS macro [62]. Hence, we tested whether transformational leadership is directly associated with team cohesion and organizational identification’s mediating role in explaining this association. Table 2 shows the standardized regression coefficients, standard errors (SE), and summary results of the hypothesized mediation model. Age, gender, and job role were included as control variables in the model.

The obtained results indicated a significant direct relationship between transformational leadership and team cohesion [b(SE) = 0.21 (0.03), *p* = 0.000, 95% CIs (0.14; 0.28)], thus supporting Hypothesis 1a. In a similar vein, transformational leadership was positively related to organizational identification [b(SE) = 0.25 (0.03), *p* = 0.000, 95% CIs (0.18; 0.31)]. This evidence provided support to Hypothesis 1b. Moreover, in line with Hypothesis 1c, the current result suggested a significant positive association between organizational identification and team cohesion [b(SE) = 0.16 (0.04), *p* = 0.003, 95% CIs (0.07; 0.23)]. Finally, the hypothesized indirect effect was confirmed (H1d) [b(SE) = 0.04 (0.01), 95% CIs (0.01; 0.07)].

Regarding our second hypothesis, the moderated mediation model was assessed using model 58. To be specific, we assume the emotional contagion moderates both the relationship between transformational leadership (i.e., the independent variable) and organizational identification (i.e., mediator) and the association between organizational identification and team cohesion (i.e., the criterion variable).

**Figure 2:**
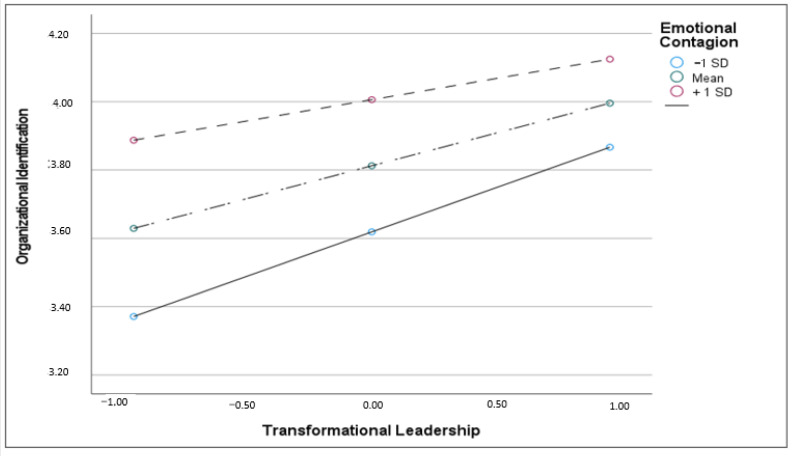
Interaction effect between transformational leadership and emotional contagion on organizational identification.

**Figure 3:**
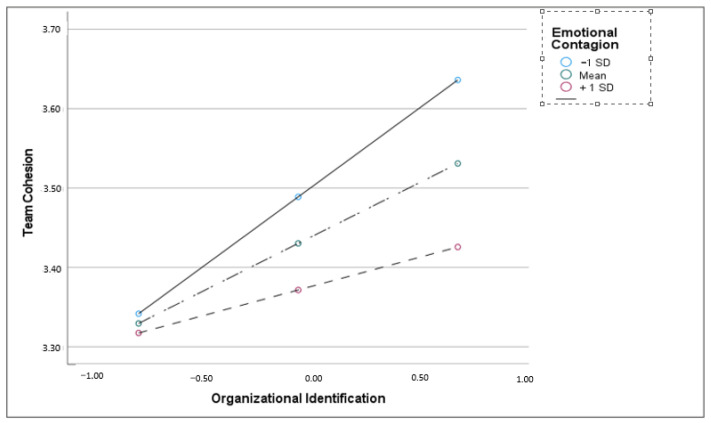
Interaction effect organizational identification and emotional contagion on team cohesion.

As Table 3 shows, in model 1 (H2a), there was a significant main effect of the leadership on organizational identification, [b(SE) = 0.20 (0.03), *p* = 0.000, CIs (0.131; 0.260)], and this effect was moderated by emotional contagion [b(se) = −0.12 (0.04), *p* = 0.003, CIs (0.004; 0.015)]. As can be seen from Figure 2, the highest levels of organizational identification occur when a high perception of transformational leadership interacts with a low susceptibility to emotional contagion. Regarding H2b, model 2 showed that the effect of organizational identification on team cohesion was significant [b(SE) = 0.13 (0.04), *p* = 0.035, CIs (0.043; 0.219)], and this effect was moderated by emotional contagion, [b(SE) = −0.11 (0.03), *p* = 0.010, CIs (−0.192; −0.024)]. In this case, as can be seen from Figure 3, the highest values of team cohesion are obtained with the highest levels of organizational identification, in interaction with the lowest levels of emotional contagion. Finally, simple slope tests showed that the indirect effect between transformational leadership and team cohesion via organizational identification was significant at low [b(se) = 0.05 (0.02), (CIs 0.020; 0.09)] and average levels of emotional contagion [b(se) = 0.03 (0.01), (CIs 0.005; 0.050)], but not at the high level of emotional contagion. Therefore, Hypothesis H2c was partially supported.

## 4. Discussion

Our study aimed to explore the association between transformational leadership and team cohesion via organizational identification considering the moderating effect of emotional contagion.

### 4.1. Direct and Mediation Effect

Regarding H1a, the current study provided support to the direct relationship between leadership and team cohesion. Cohesion, similar to cooperation among employees toward a common goal, has been considered one of the primary behaviors that characterize transformational leaders since the earliest conceptualizations in the field [33]. Transformational leaders are likely to increase team cohesion by encouraging their followers to overcome their own interests in favor of the interests of the group as a whole [65]. Moreover, previous findings show that transformational leadership is a direct antecedent of workplace well-being [66] and that cohesion is a mechanism involved in this relationship [67].

Our results also confirmed H1b, indicating a positive relationship between transformational leadership and organizational identification. This finding is consistent with research that has shown a relationship between organizational identification and transformational leadership style [34]. According to the innovative study by Kark and Shamir [37], transformational leaders are able to foster two distinct self-concepts among employees: the relational self, encouraging followers to identify individually with their leader, and the collective self, involving stronger social identification with one’s organization as a whole. Transformational leaders strengthen employees’ commitment to the organization by motivating, empowering, and supporting their followers [36], which reinforces the sense of belonging and the notion of being part of something bigger [38].

Moreover, organizational identification was associated with higher levels of team cohesion in our sample, confirming H1c. Since organizational identification is responsible for making employees feel connected to their organization [40], belonging to a particular group provides a sense of identity. Thus, when employees identify strongly with the organization, they are more likely to internalize its norms and goals and to view themselves as interchangeable with other members. Such stronger identification should then lead to better cooperation among employees [39].

Another finding is that identification with the organization is a mediator in the relationship between transformational leadership and team cohesion (H1d). The key components of transformational leadership and its effects on team cohesion and organizational identification could be responsible for this result. Fostering employees’ collective self-identity increases the likelihood that they will behave cooperatively to support the organization’s mission and goals rather than their personal goals [68].

### 4.2. Moderation Effect

Regarding our second hypothesis, we found conflicting results with the limited available literature on emotional contagion and the variables considered in the current study. According to previous research by Vijayalakshmi and Bhattacharyya [11] emotional contagion is positively related to team cohesion, transformational leadership, and leadership outcomes [52,69]. In particular, the review proposed by Tee [48] concluded that implicit and explicit emotional contagion processes have an important impact on organizational leadership outcomes, which highlights the importance of contagion processes in explaining emotional connections between different levels of organizations [54]. Based on previous empirical evidence and theoretical arguments, we expected that the relationship between transformational leadership and organizational identification should be even stronger at high levels of employee emotional contagion.

We also found higher levels of organizational identification among employees when characterized by low levels of emotional contagion. These results are not consistent with the previous scientific literature. Previous results indicate that emotional contagion has a positive effect on organizational identification and promotes better integration within the organization. Our results also show that team cohesion was higher when emotional contagion was low and medium. Moreover, the indirect relationship between transformational leadership and team cohesion via organizational identification was stronger when employees reported low and medium levels of emotional contagion.

Our results could be interpreted in terms of a so-called “compensatory effect”. Accordingly, for teachers who report high levels of emotional contagion and are already very sensitive to the emotions of their colleagues, the (transformational) leadership style of their principal is less relevant (lack of reference to organizational identification, control). On the other hand, a transformational leadership style, which also aims to recognize and empathize with the other, including their emotions, helps teachers who are less likely to compensate for this deficit.

In addition, researchers found that those who are more vulnerable to emotional contact are more prone to depersonalization and a diminished sense of personal fulfillment, which contributes to emotional fatigue [70] and, consequently, lower well-being. Other studies [71,72] have shown that nurses who shared their patients’ feelings more frequently were more likely to experience emotional exhaustion. Therefore, we can hypothesize that in our sample of teachers, the principal’s leadership skills are no longer sufficient to strengthen team cohesion when emotional contagion is too high, as they generally report high levels of exhaustion, even when mediated by identification with the organization that leads to shared values and vision. In addition, we identified one study in the literature that hypothesized a detrimental moderating effect for emotional contagion. Xerri and colleagues [71] hypothesized that emotional contagion negatively affects the relationship between psychological capital and employee well-being, such that employees with high levels of emotional contagion experience a lower impact of psychological capital than employees with low levels of emotional contagion. Finally, they hypothesized that high levels of emotional contagion could have a detrimental effect on the impact of a (personal) resource on personal well-being. In other words, people who are highly susceptible to emotional contagion are unable to control their own emotions when confronted with those of others, which can lead to a reduced sense of team cohesion, which subsequently could lead to reduced perceived well-being and decreased performance.

Our study is not without limitations. First, the study was conducted among Italian participants, which prevents generalization of the current results to other cultural and social contexts. Future studies should aim to include participants from diverse regions and countries to assess the robustness of the observed associations across different contexts. Moreover, this self-report cross-sectional study does not allow us to hypothesize random relationships between variables, but only to observe associations. Future studies should use a longitudinal study design, also integrating objective measures (e.g., student grades; daily surveys of teachers’ stress levels), so as to show relationships among the variables investigated, also adding information with respect to the influence of transformational leadership on teachers’ performance and well-being. Nonetheless, our findings attempt to fill the gap in the literature regarding the role of emotional contagion in educational contexts and highlight practical implications.

Our study significantly contributes to understanding the positive outcomes of transformational leadership in schools. Transformational leaders foster teachers’ identification with their school, enhancing group cohesion, which, in turn, yields various positive outcomes at individual and organizational levels, (e.g., well-being [71]). Group cohesion is highly desirable for educational organizations as it facilitates communication, collaboration, and improved teacher performance. Additionally, a cohesive group reduces the risk of social isolation, particularly important for early career teachers. Transformational leaders increase followers’ sense of belonging, even among those with low emotional contagion levels, leading to increased team cohesion.

Schools should invest in leadership development programs to cultivate leadership skills in teachers [73]. These programs should emphasize commitment to the school’s mission and vision, encourage professional development, support innovative ideas, and enhance communication abilities through feedback and rewards. Formalized mentoring programs can also be beneficial, providing support and guidance to teachers facing educational challenges ([74] p. 3). Creating a shared culture with a clear mission can increase school identification, fostering reciprocal support among teachers in dealing with high workloads [39]. Finally, our findings show a role as a moderator played by emotional contagion. Contrary to our expectations and the existing literature, we did not find an additive effect of emotional contagion. The effect of a transformational leadership in fostering a strong sense of belonging and cohesiveness among followers was stronger for teachers with low levels of emotional contagion. These findings should be replicated in other samples and by using other research designs, before drawing conclusions. Anyway, these findings suggest to us that individuals with more difficulties in understanding and consistently reacting to the emotions of others are also the ones who benefit most from a positive leadership style and a shared sense of belonging. These findings reinforce the importance for the schools to engage in a leadership developmental program and create a common school identity. Initiatives promoting conscious management of emotions in a stressful environment can help prevent burnout symptoms. Open communication about feelings and emotions should be encouraged to support teachers in recognizing, managing, and enhancing their emotional abilities. In this sense, the results of this study may be useful for educational institutions, as they highlight the importance of training leaders and teachers on the importance of effective leadership styles and strategies for emotional regulation, understanding the emotions of others, and effectively expressing one’s own emotions.

## 5. Conclusions

To date, the challenges and changes faced by teachers are intense. Several studies have shown how the skills of school leaders can help improve teachers’ personal and professional outcomes. In addition, recent studies have shown the importance of organizational identification, i.e., the common values that teachers share with the organization to which they belong. The role of emotions in such relationships has often been neglected. Recently, studies on emotional contagion have increased, but they still seem inconclusive about the relationships between collective and individual variables. Until now, to our knowledge, no study had considered such relationships among the variables examined. We sought to fill the gap in the literature with respect to studies aimed at clarifying the link between transformational leadership and group outcomes, as well as enlightening the moderating role of emotional contagion between leadership, organizational identification, and team cohesion. Our study demonstrates the positive role of a transformational leadership style in fostering strong organizational identification within the school context, with only limited prior evidence on this aspect. Additionally, our investigation delves into the relationship between identification and group cohesion.

Unexpectedly, our findings regarding emotional contagion revealed a moderation effect consistent for low levels, rather than high levels, of emotional contagion. This novel result represents a significant contribution of our paper, shedding new light on this aspect. School leaders who cultivate a transformational leadership style are able to foster teacher identification with their organization, which in turn leads to greater team cohesion. However, this relationship becomes irrelevant at high levels of emotional contagion, underscoring the importance of providing adequate resources and opportunities for teachers to actively share their emotions without the risk of being overwhelmed.

## Figures and Tables

**Table 1:** Means, SDs, correlation, and reliability of variables included in the study.

	Mean/Freq	SD	1.	2.	3.	4.	5.	6.
Transformational Leadership	3.71	0.93	(0.94)					
Organizational Identification	3.80	0.77	0.31 **	(0.85)				
Team Cohesion	3.42	0.73	0.34 **	0.25 **	(0.83)			
Emotional Contagion	3.74	0.56	0.19 **	0.36 **	0.06	(0.86)		
Gender	72.1% (F)	0.45	0.10 *	0.09 *	−0.01	0.24 **	-	
Age	47.06	11.42	0.04	0.22 **	0.07	0.20 **	−0.01	-
Work Role	82.4% (II)	0.49	−0.02	0.01	0.01	0.01	−0.05	0.06

Note: Cronbach’s on the diagonal. ** *p* < 0.01; * *p* < 0.05; *SD* = standard deviation; gender: 0 = male, 1 = female; work role: II = high school.

**Table 2:** Indirect effects for mediating effects.

Indirect Effect	Est.	SE	95% CI
Transformational leadership → Organizational identification → Team cohesion	0.04	0.01	(0.01, 0.07)

Note: All parameter estimates are presented as standardized coefficients. Estimates (Est.). Standard error (SE). Confidence interval (95% CI).

**Table 3:** Testing the moderated mediation effect of the emotional contagion on transformational leadership on team cohesion.

	Model 1 (to Organizational Identification)		Model 2 (to Team Cohesion)	
	B (SE)	t	B (SE)	t
Gender	0.06 (0.07)	0.83	−0.07 (0.07)	−0.95
Age	0.01 ** (0.03)	3.62	0.00 (0.00)	0.58
Work Role	0.01 (0.06)	0.02	−0.01 (0.06)	−0.12
LEAD	0.19 *** (0.03)	6.03	0.21 *** (0.03)	6.06
EMO	0.35 *** (0.06)	6.07	−0.11 (0.06)	−1.72
LEAD × EMO	−0.12 ** (0.04)	−3.13	-	-
ORG ID	-	-	0.13 ** (0.05)	2.93
ORG ID × EMO	-	-	−0.11 * (0.42)	−2.53
R^2^	0.01		0.01	
F	9.79		6.40	

Note: All parameter estimates are presented as standardized coefficients. *** *p* < 0.001; ** *p* < 0.01; * *p* < 0.05 Estimates (Est.). Standard error (SE). Confidence interval (CI). Transformational Leadership (LEAD). Organizational Identification (ORG IDENT). Team Cohesion (COHESION).

## Data Availability

Data used for the study are available from the corresponding author upon reasonable request.
